# Interdependency and phosphorylation of KIF4 and condensin I are essential for organization of chromosome scaffold

**DOI:** 10.1371/journal.pone.0183298

**Published:** 2017-08-17

**Authors:** Rawin Poonperm, Hideaki Takata, Susumu Uchiyama, Kiichi Fukui

**Affiliations:** 1 Department of Biotechnology, Graduate School of Engineering, Osaka University, Suita, Osaka, Japan; 2 Biomedical Research Institute, National Institute of Advanced Industrial Science and Technology (AIST), Ikeda, Osaka, JAPAN; 3 Chromosome Engineering Research Center, Tottori University, Yonago, Japan; Institut de Genetique et Developpement de Rennes, FRANCE

## Abstract

Kinesin family member 4 (KIF4) and condensins I and II are essential chromosomal proteins for chromosome organization by locating primarily to the chromosome scaffold. However, the mechanism of how KIF4 and condensins localize to the chromosome scaffold is poorly understood. Here, we demonstrate a close relationship between the chromosome localization of KIF4 and condensin I, but not condensin II, and show that KIF4 and condensin I assist each other for stable scaffold formation by forming a stable complex. Moreover, phosphorylation of KIF4 and condensin I by Aurora B and polo-like kinase 1 (Plk1) is important for KIF4 and condensin I localization to the chromosome. Aurora B activity facilitates the targeting of KIF4 and condensin I to the chromosome, whereas Plk1 activity promotes the dissociation of these proteins from the chromosome. Thus, the interdependency between KIF4 and condensin I, and their phosphorylation states play important roles in chromosome scaffold organization during mitosis.

## Introduction

DNA with histone proteins, known as chromatin, remains relaxed during the G_1_-G_2_ stages of the cell cycle. Initiation of mitosis leads the chromatin condensation and the chromatin remains compact until the end of mitosis. Although the chromosome scaffold is first visualized during the initial period of the middle-late prophase and becomes more distinct during the prometaphase to metaphase [1], some scaffold proteins have been found to localize to chromatin prior to the initiation of chromosome condensation or during interphase. Condensin II, topoisomerase Iiα (Topo IIα) and kinesin family member 4 (KIF4) interact with chromatin during interphase and form the chromosome scaffold during mitosis [2, 3]. Topo IIα localizes at chromosome axial positions during prophase, suggesting Topo IIα plays a functional role in guiding chromosome scaffold construction and chromosome condensation [1, 4]. Condensin II also localizes to chromosome axes during the early prophase and may play a primary role in the formation of the chromosome scaffold [5, 6]. It is previously shown that KIF4 involves in cytokinesis by functioning at midzone during cell division [7]. In addition, several reports suggest that KIF4 also contributes to the higher order structure of chromatin and condensation of chromosome [2, 8–11]. Condensin I is another major scaffold protein that is located primarily in the cytoplasm during G_1_, S and G_2_ stages. Consequently, condensin I is loaded to the chromosome after nuclear envelop breakdown [6, 12–14]. Recently, double-stranded scaffold structure was observed in the metaphase chromosome by 3D-SIM and depletion of either scaffold protein caused defects in the scaffold structure [10]. The correct assembly of scaffold proteins therefore provides the foundation for proper formation of the chromosome scaffold and chromosome morphology [9, 10]. Thus, regulation of scaffold protein localization to chromosomes is essential for construction of the chromosome scaffold. However, the mechanism that regulates the recruitment of these proteins to the chromosome scaffold remains unknown.

Multi-regulatory factors and several protein modulators are reported to control association of scaffold proteins to the chromosome. In *Xenopus*, Protein phosphatase 2A (PP2A) acts as a direct recruiter of condensin II and KIF4 to the chromosome by noncatalytic function [15]. In addition, condensin II is also recruited to the chromosome by retinoblastoma protein in humans [16]. In *S*. *pombe*, monopolin acts as a recruiter of condensin to the centromere [17]. Sgo1 also physically directs condensin to be enriched on the centromere in budding yeast [18]. These observations indicate that recruitment of scaffold proteins to chromatin or the chromosome scaffold is mediated by recruiters that are not identified yet.

Post-translational modifications of proteins are essential for the association of scaffold proteins to the chromosome. The condensin II complex associates with chromatin in interphase, and Cdk1-mediated phosphorylation of a condensin II subunit, CAP-D3, is thought to trigger proper chromosome condensation [19, 20]. The phosphorylation of hCAP-H2 at S492 by Mps1 also regulates condensin II loading to the chromosome [21]. After nuclear envelope breakdown, condensin I binds to the chromosome by Aurora B-dependent phosphorylation [22–25]. Aurora B phosphorylates hCAP-H, a non-SMC subunit of condensin I, and regulates condensin I loading to the chromosome [22, 25]. Two more phosphorylation sites on hCAP-G, another non-SMC subunit of condensin I, were found to control condensin I binding to the chromatin [26]. Cdc2 phosphorylation is important for chromatin binding of condensin I in *Xenopus* [27].

Regulation of KIF4 loading to the chromosome remains poorly understood. Nonetheless, recent results showed that KIF4 and condensin phosphorylation by Cdk1 and Aurora B are required for the interaction between KIF4 and condensin [28] and the motor activity of KIF4 might be involved in the proper localization of condensin I at chromosome scaffold [11]. In this study, we further examined the interdependency of KIF4 and other scaffold proteins in chromosome organization. The results reveal that KIF4 localization to the chromosome scaffold is regulated interdependently with condensin I, but not with condensin II. Moreover, interaction between KIF4 and condensin I is involved in establishing the chromosome scaffold, and Aurora-B- and Plk1-dependent phosphorylation of KIF4 and condensin I regulates association and dissociation of KIF4 and condensin I to and from chromosomes, respectively.

## Materials and methods

### Cell culture

HeLa cells were obtained from Yamamoto Lab. at the Institute of Medical Science the University of Tokyo. HEK293T cells were obtained from Maenaka Lab. at Faculty of Pharmaceutical Sciences Hokkaido University. These cells were cultured in a complete medium, Dulbecco’s modified Eagle’s medium (DMEM; GIBCO BRL) supplemented with 10% fetal-bovine serum (FBS; Equitech-Bio), at 37°C and 5% CO_2_. Stable HeLa cells expressing GFP tagged hCAP-D3 [20] were maintained selected in the complete medium containing 0.2 mg/ml puromycin (SIGMA). Stable HEK293T cells expressing GFP tagged hCAP-H-wt or GFP tagged hCAP-H-S70A were maintained selected in the complete medium containing 1 mg/ml puromycin (SIGMA).

### Synchronization

At 10–15% confluence, HeLa or HEK293T cells were synchronized in the complete medium containing 2.5 mM final concentration of thymidine (SIGMA). After first thymidine blocking was applied for 24 hr, thymidine was removed by washing twice with PBS, then the cells were subjected to culture in the fresh complete medium again for 12 hr to release cells. HeLa cells were synchronized in the complete medium containing thymidine (final concentration 2.5 mM) for 18 hr. After second thymidine blocking, thymidine was removed by washing twice with PBS, then the cells were subjected in the fresh complete medium again to release cells. To obtain mitotic cells, cells were released in the presence of 100 ng/ml nocodazole for 12 hr before harvesting.

### Cell and chromosome immunostaining and fluorescence microscopy observation

Cells were treated with a hypotonic solution, 75 mM KCl for 15 min at 37°C then spun onto coverslip (ZEISS) coated with poly-L-lysine (SIGMA) by cytocentrifugation (Shandon Cytospin 4, Thermo) at 1,300 rpm for 10 min. Then, cells were fixed with 2% PFA (*para*-formaldehyde) in PBS for 15 min, or with iced-cold 100% methanol at -20°C for 15 min, and permeabilized by 0.2% Triton X-100 in PBS for 10 min. Cells were blocked with 3% BSA in PBS for 1 hr then primary and secondary antibody reactions were performed under the same condition, at room temperature for 1 hr, respectively. Samples were mounted in Vectorshield mounting medium (Vector Laboratories). DNA was counterstained by Hoechst 33342 or DAPI. Samples were visualized by fluorescence microscopy, Axioplan 2 (ZEISS) or DeltaVision (GE Healthcare).

### Preparation of ZM447439- or BI2536-treated cells

HeLa cells were synchronized by thymidine block and release in medium containing 100 ng/ml nocodazole for 14 hr in the presence of 4μM ZM447439 or 100 nM BI3536 during the last 2hr. Mitotic cells were collected by mitotic shake-off.

### RNA interference

The small interfering RNA (siRNA) sequences were as follows,

KIF4, 5′-GCAAUUGAUUACCCAGUUATT-3′;

SMC2, 5′-UGCUAUCACUGGCUUAAAUTT-3′;

hCAP-D2, 5′-CCAUAUGCUCAGUGCUACATT-3′,

hCAP-D3, 5′-CAUGGAUCUAUGGAGAGUATT-3′

For transfection, HeLa cells were incubated with 120–200 nM siRNAs using Lipofectamine^®^2000 (Invitrogen). Transfection was performed concomitantly with synchronization. After 72 hr of incubation, cells were collected and used for analysis.

### Cell or chromatin extraction and western blot

Whole cell lysates were obtained by direct re-suspension of mitotic cells in 2×Laemmli sample buffer before sonication. For chromatin extraction, mitotic cells were collected and washed by PBS once. Cell pellets were re-suspended in freshly prepared CSK1 buffer (10 mM PIPES, pH 6.8, 10% glycerol, 3 mM MgCl_2_, 100 mM NaCl, 1 mM DTT, 0.25 mM PMSF, 0.3% triton X-100) for 30 min on ice. Insoluble chromatin pellets were collected by centrifuging at 16,100 *g* for 10 min and washed again with CSK1 buffer. The chromatin pellets were re-suspended 2×Laemmli sample buffer before sonication. Western blotting was performed as described below. Extracted proteins from cell lysates and chromatin fractions were separated by SDS-PAGE and transferred to a membrane using iBlot (Invitrogen) or conventional electroblotting. Membrane blocking was performed in 2% BSA in TBST (20 mM Tris, pH 7.5, 150 mM NaCl, 0.1% (v/v) Tween 20). Primary and secondary antibodies were also diluted in 2% BSA in TBST. Primary antibody reaction was performed at 4°C for overnight and secondary antibody reaction was performed at room temperature for 1 hr. Immunoreactive bands of proteins were detected using 1% NBT/BCIP stock solution (Roche) in alkaline phosphatase buffer (100 mM Tris, pH 9.5, 100 mM NaCl, 5 mM MgCl_2_).

### Preparation of chromatin-bound or chromatin-unbound protein fractions

HeLa or HEK293T cells were synchronized by thymidine block and release in medium containing 100 ng/ml nocodazole. Mitotic cells were collected (1–2×10^7^ cells/reaction) and washed by PBS once. Cell pellets were re-suspended in CSK1 buffer (10 mM PIPES, pH 6.8, 10% glycerol, 3 mM MgCl_2_, 100 mM NaCl, 1 mM DTT, 0.25 mM PMSF, 0.3% triton X-100) for 30 min on ice. Soluble fraction was collected as chromatin-unbound fraction or cytoplasmic fraction. Insoluble chromatin pellets were collected by centrifuging at 16,100 *g* for 10 min and washed again with CSK1 buffer. The chromatin pellets were then re-suspended in CSK2 buffer (10 mM PIPES, pH 6.8, 10% glycerol, 3 mM MgCl_2_, 250 mM NaCl, 2.5 mM CaCl_2_, 1 mM DTT, 0.25 mM PMSF) containing 20 units MNase (Takara) and incubated at 37°C for 30 min. After centrifugation at 16,100 *g* for 10 min, supernatant was collected as chromatin-bound protein fraction. Note: All CSK buffers contained complete mini protease inhibitor cocktail (Roche) and phosphatase inhibitor PhosSTOP (Roche) and were freshly prepared.

### Immunoprecipitation

The chromatin-bound or chromatin-unbound fractions were rotated with specific antibody against the targeting protein (1 μl/sample) or normal rabbit IgG as control (1 μl/sample) for overnight at 4°C, followed by 2–4 hr rotation with protein A sepharose beads at 4°C. The beads were washed three times with CSK3 buffer (10 mM PIPES, pH 6.8, 10% glycerol, 3 mM MgCl_2_, 250 mM NaCl, 1 mM DTT, 0.25 mM PMSF). Immunoprecipitates were analyzed by western blot. Note: All CSK buffers contained protease inhibitor and phosphatase inhibitor and were freshly prepared.

### Antibodies

List of antibody used in the experiments were shown in S1 Table.

### Plasmid, plasmid transfection and stable cell line generation

Two expression plasmids, pEGFP-hCAP-H-wt and pEGFP-hCAP-H-S70A were obtained from Watanabe lab, University of Tokyo [25]. HEK293T cells were seeded at 30% confluent in 24-well plate one day before transfection. Then, pEGFP-hCAP-H-wt or pEGFP-hCAP-H-S70A were transfected into the cells using 4:1, X-tremeGENE HP DNA Transfection Reagent: Plasmid ratio, according to manufacturer’s protocol. At 48 hr post-transfection, transfection efficiency was evaluated by direct observation using fluorescence microscopy. Puromycin was used for the stable cell line establishment and its concentration was reduced after 3–4 weeks.

## Results

### KIF4 localization at the chromosome scaffold is regulated by the interaction with condensin I, but not condensin II

Previous reports revealed a close relationship between KIF4 and condensin, and their role in chromosome condensation [8–11]. However, discrepancies in their interdependency for the chromosome localization among these previous studies existed. Mazumdar *et al*. found that KIF4 interacts with both condensin I and II, and a knockdown of KIF4 did not change the total condensin level on chromosomes in human cells [8]. In contrast, using proteome analysis Samejima *et al*. revealed a relationship between KIF4 and condensin I, but not condensin II, to their chromosome localization in DT40 cells by showing a significant reduction of condensin I on chromosomes in KIF4-depleted DT40 cells [9].

**Fig 1:**
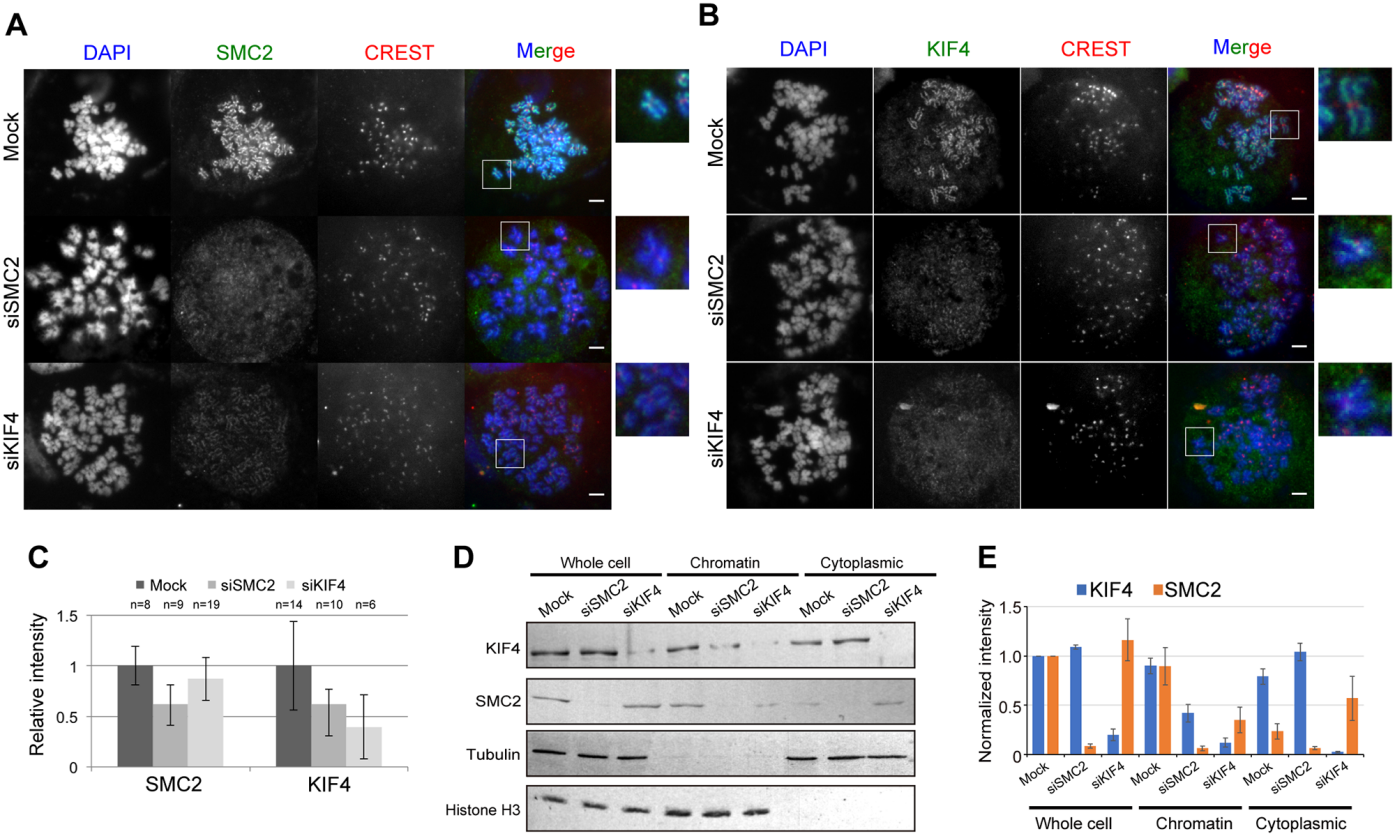
Localization of condensin and KIF4. (A) Mock, KIF4-depleted or SMC2-depleted HeLa cells were immunostained against SMC2 and CREST. DNA was counter stained with DAPI. Scale bar, 5 μm. Close-ups of the regions indicated by white boxes were shown on the right. (B) Mock, KIF4-depleted or SMC2-depleted HeLa cells were immunostained against KIF4 and CREST. DNA was counter stained with DAPI. Scale bar, 5 μm. Close-ups of the regions indicated by white boxes were shown on the right. (C) Quantification of relative mean fluorescence intensity of SMC2 or KIF4 staining in cells transfected with mock, SMC2- or KIF4-siRNA as indicated. Fluorescence intensities are normalized to the average pixel intensity of mock-transfected cells from the same experiment. Error bars represent the standard deviation. Two independent experiments were performed and data shown are from one representative experiment. (D) Western blot analysis of whole cell extract, chromatin fraction and cytoplasmic fraction prepared from mitotic mock, SMC2-depleted or KIF4-depleted HeLa cells. Tubulin and histone H3 were used as loading controls. (E) The band intensity of (D) was quantified using ImageJ software and normalized against protein amount in whole cell extract of mock. n = 3.

To resolve these results, we first depleted KIF4 or SMC2 (a subunit of condensin) from HeLa cells and determined the amount of scaffold proteins on chromosomes. We revealed that the level of SMC2 on chromosomes was reduced in the absence of KIF4 (Fig 1A and 1C–1E). In parallel, the level of KIF4 was also reduced on chromosomes in the absence of SMC2 or condensin (Fig 1B and 1C–1E). These results confirmed the functional relationship between KIF4 and condensin for loading to chromosomes, and the results are consistent with the previous report obtained using DT40 cells [9].

Mammalian cells have condensin I and II [12]. Because we confirmed a reduction of the condensins levels on chromosomes by depletion of KIF4, and *vice versa* (Fig 1), we further examined whether the level of condensin I or II or both proteins on chromosomes were affected by the depletion of KIF4 in HeLa cells expressing GFP-tagged hCAP-D3 (HeLa-GFP-hCAP-D3).

**Fig 2:**
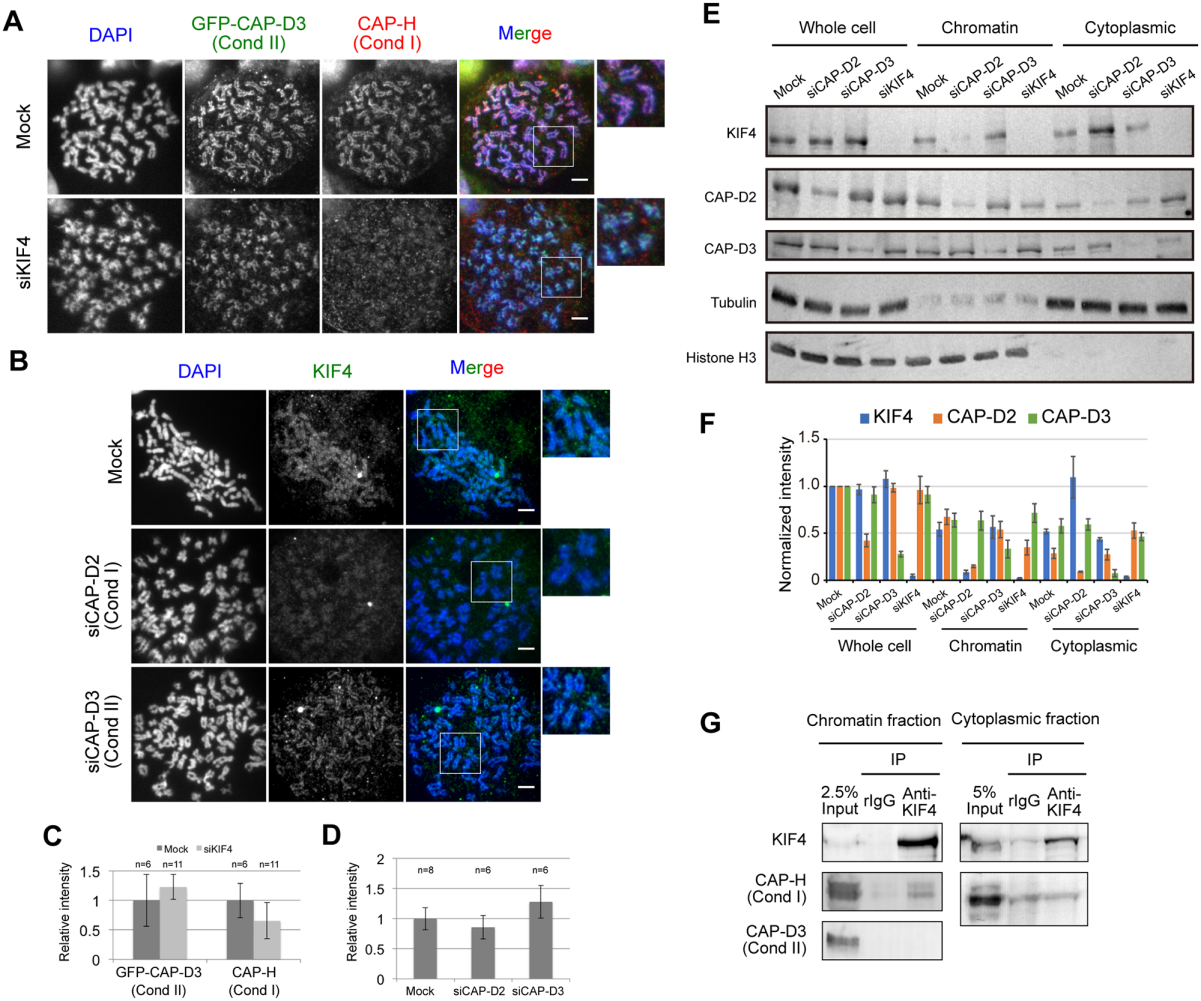
Interdependency between KIF4, condensin I and II. (A) Localization of hCAP-H (condensin I) and GFP-hCAP-D3 (condensin II) in mock- or KIF4-depleted HeLa cells were confirmed by immunostaining. DNA was counter stained with DAPI. Scale bar, 5 μm. Close-ups of the regions indicated by white boxes were shown on the right. (B) Localization of KIF4 in mock, hCAP-D2-depleted or hCAP-D3-depleted HeLa cells were confirmed by immunostaining. DNA was counter stained with DAPI. Scale bar, 5 μm. Close-ups of the regions indicated by white boxes were shown on the right. (C) Quantification of relative mean fluorescence intensity of GFP-hCAP-D3 or hCAP-H staining in cells transfected with mock or KIF4-siRNA as indicated. Fluorescence intensities are normalized to the average pixel intensity of mock-transfected cells from the same experiment. Error bars represent the standard deviation. Two independent experiments were performed and data shown are from one representative experiment. (D) Quantification of relative mean fluorescence intensity of KIF4 staining in cells transfected with mock, hCAP-D2—or hCAP-D3-siRNA as indicated. Fluorescence intensities are normalized to the average pixel intensity of mock-transfected cells from the same experiment. Error bars represent the standard deviation. Two independent experiments were performed and data shown are from one representative experiment. (E) Western blot analysis of whole cell extract, chromatin fraction and cytoplasmic fraction prepared from mitotic mock, hCAP-D2-depleted, hCAP-D3-depleted or KIF4-depleted HeLa cells. Tubulin and histone H3 were used as loading controls. (F) The band intensity of (E) was quantified using ImageJ software and normalized against protein amount in whole cell extract of mock. At least three independent experiments were performed. (G) Physical interaction between KIF4, condensin I and condensin II. KIF4 was immunoprecipitated from chromatin-bound or chromatin-unbound protein fractions. Co-immunoprecipitation of hCAP-H (condensin I) and hCAP-D3 (condensin II) with KIF4 was analyzed by western blot. n = 3.

After KIF4 depletion, we found that endogenous hCAP-H and hCAP-D2 (a subunit of condensin I) were reduced on chromosomes, whereas GFP-hCAP-D3 and endogenous hCAP-D3 did not change (Fig 2A, 2C, 2E and 2F). These results are consistent with the study by Samejima *et al*. [9], indicating that the absence of KIF4 affected the loading ability of condensin I, but not condensin II, to the chromosome.

To further understand the relationship between KIF4 and condensin I, reciprocal experiments were conducted. Condensin I and II were individually depleted from HeLa-GFP-hCAP-D3 using siRNA targeting hCAP-D2 or hCAP-D3, respectively. Then KIF4 localization was observed. We found that KIF4 was reduced on the chromosomes in hCAP-D2 depleted cells (Fig 2B, 2D, 2E and 2F). In contrast, in hCAP-D3-depleted cells, KIF4 localization and amount on chromosome axes were slightly increased (Fig 2B, 2D, 2E and 2F), compared to mock-treated cells. These results indicate an interdependency of localization of KIF4 and condensin I on the chromosome scaffold.

Next, the physical interaction between KIF4, condensin I and II was investigated by immunoprecipitation. Chromatin-bound protein fractions were extracted from mitotic arrested HeLa cells and used for immunoprecipitation. We found that hCAP-H co-immunoprecipitated with KIF4. However, hCAP-D3 did not co-immunoprecipitate with KIF4 (Fig 2G). These data demonstrate that KIF4 interacts with condensin I, but not with condensin II. Furthermore, interaction between KIF4 and condensin I in the cytoplasmic fraction was investigated. We confirmed that hCAP-H was not co-immunoprecipitated with KIF4 in the cytoplasm (Fig 2G). These results indicate that KIF4 interacts with condensin I on the chromosome and not in the cytoplasm. Taken together, KIF4 and condensin I are closely related to each other for their localization on the chromosome.

### Inhibition of Aurora B affects loading of both KIF4 and condensin I to the chromosome

**Fig 3:**
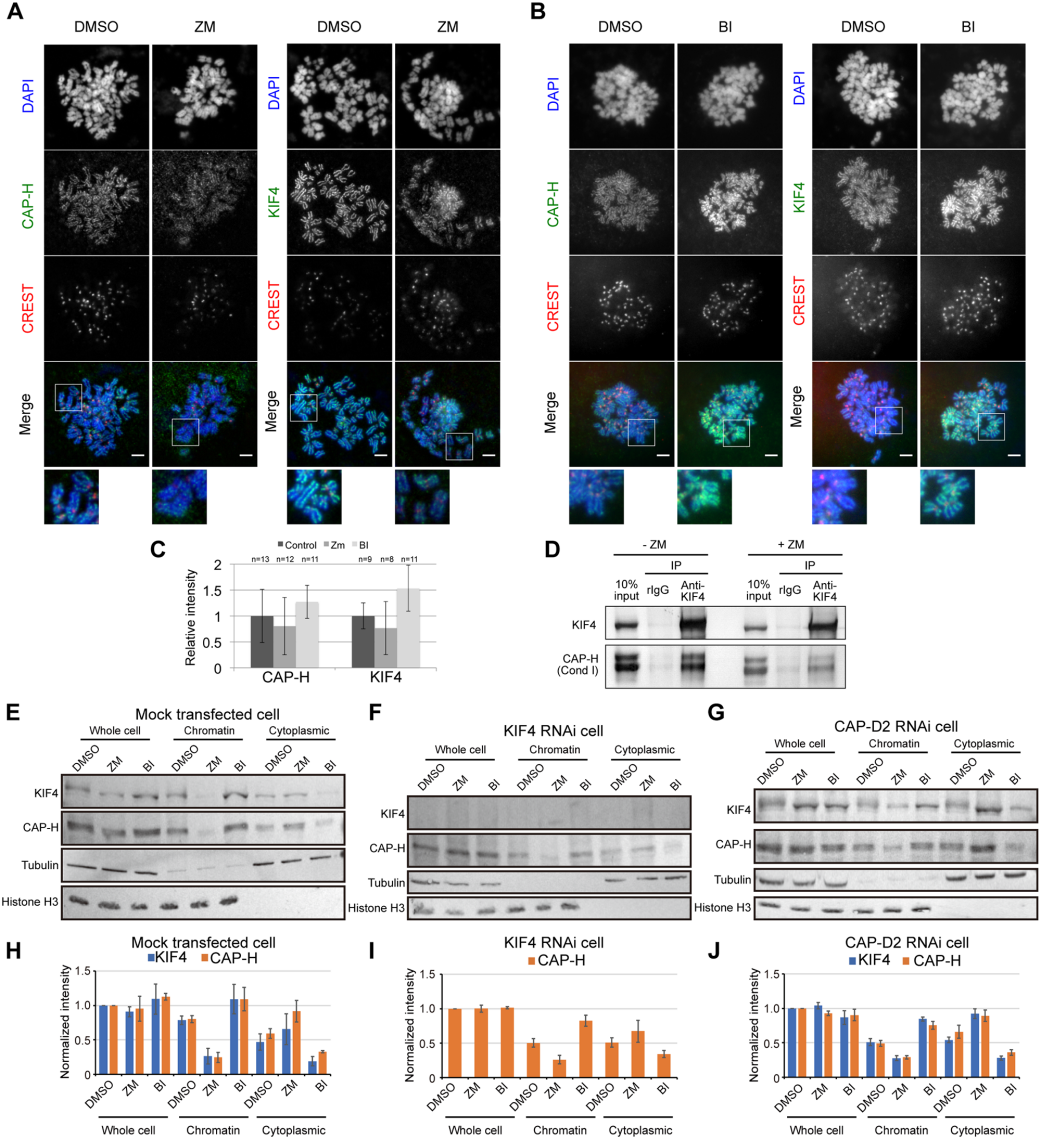
Effects of Aurora B and Plk1 inhibition on KIF4 and condensin I localization on chromosomes. (A) Immunofluorescence analysis of mitotic HeLa cells treated with DMSO as a control (DMSO) or 4 μM ZM447439 (ZM). Scale bar, 5 μm. Close-ups of the regions indicated by white boxes were shown in the bottom. (B) Immunofluorescence analysis of mitotic HeLa cells treated with DMSO as a control (DMSO) or 0.1 μM BI2536 (BI). Scale bar, 5 μm. Close-ups of the regions indicated by white boxes were shown in the bottom. (C) Quantification of relative mean fluorescence intensity of hCAP-H or KIF4 antibody in cells treated with DMSO, 4 μM ZM447439 (ZM) or 0.1 μM BI2536 (BI) as indicated. Fluorescence intensities are normalized to the average pixel intensity of DMSO-treated cells from the same experiment. Error bars represent the standard deviation. At least, three independent experiments were performed and data shown are from one representative experiment. (D) Physical interaction between KIF4, condensin I and condensin II. KIF4 was immunoprecipitated from chromatin-bound protein fractions extracted from mitotic HeLa cells treated without or with 4 μM ZM447439 (-ZM and +ZM, respectively). Co-immunoprecipitation of hCAP-H with KIF4 was analyzed by western blot. (E), (F) and (G) Western blot analysis of whole cell extract, chromatin fraction and cytoplasmic fraction prepared from mitotic mock, KIF4-depleted or hCAP-D2-depleted HeLa cells, respectively. The cells were treated with DMSO or 4 μM ZM447439 (ZM) or 0.1 μM BI2536 (BI). Tubulin and histone H3 were used as loading controls. (H), (I) and (J) The band intensity of (E), (F) and (G), respectively, was quantified using ImageJ software and normalized against protein amount in whole cell extract of mock. n = 3.

It has been shown that condensin I loading to the chromosome is largely regulated by Aurora B [22–25]. This modification of condensin I is found to be conserved from yeast to humans [25]. Interdependency and physical interaction between KIF4 and condensin I for their chromosome localization raise the question whether KIF4 is regulated by Aurora B. Thus, whether Aurora B kinase inhibition would affect KIF4 loading to the chromosome was examined using HeLa cells synchronized and arrested by nocodazole in the absence or presence of Aurora B kinase inhibitor, ZM447439 (ZM). Reduced levels of hCAP-H on the chromosome were observed under Aurora B kinase inhibition (Fig 3A, 3C and 3E–3H). This result is consistent with the previous reports [22–25]. Furthermore, KIF4 levels on chromosomes were also reduced under Aurora B kinase inhibition (Fig 3A, 3C and 3E–3H)., consistent with the results obtained for condensin I. However, there is a small amount of hCAP-H and KIF4 remained in chromatin fractions after ZM treatment, suggesting that Aurora B partly promotes loading of condensin I and KIF4 to the chromosomes, but there might be other pathways to compensate condensin I loading to chromosome in the absence of phosphorylation by Aurora B.

Depletion of KIF4 resulted in a decrease in the amount of condensin I on chromosomes and depletion of condensin I caused a reduction in KIF4 levels (Fig 2A–2F). In addition, Aurora B inhibition reduced both KIF4 and condensin I levels on chromosomes (Fig 3A, 3C and 3E–3H). We next examined whether the reduction of KIF4 and condensin I on chromosomes by the inhibition of Aurora B was more pronounced for either of the proteins. We found that reduction of hCAP-H on chromosomes was more distinct by ZM treatment than the KIF4 knockdown (Fig 3E, 3F, 3H and 3I). However, depletion of KIF4 in the presence of ZM did not show greater reduction of hCAP-H when compared with that of the mock with ZM treatment (Fig 3E, 3F, 3H and 3I). Knockdown of hCAP-D2, a subunit of condensin I in the presence of ZM also showed consistent results which reduction of KIF4 on chromosome was more distinct in the ZM treatment than hCAP-D2 knockdown. And in the presence of ZM, KIF4 levels were similar between mock and hCAP-D2 depleted cells (Fig 3E, 3G, 3H and 3J). These results suggest that interdependency and phosphorylation would consist a part of the same pathway of KIF4 and condensin I localization to the chromosome, and that phosphorylation is an upstream event for their binding to the chromosome because a reduction of protein amount is dominant in ZM treated cells than knockdown cells.

### Interaction between KIF4 and condensin I is independent of phosphorylation by Aurora B

We hypothesized that KIF4 and condensin I interaction would assist in the localization on the chromosome scaffold. Furthermore the interaction between KIF4 and condensin I might be promoted by phosphorylation by Aurora B kinase. To examine this hypothesis, the interaction between KIF4 and condensin I in the absence or presence of ZM was investigated. Mitotic arrested HeLa cells were incubated either in the absence or presence of ZM. Chromatin-bound protein fractions were extracted from these cells and were used for immunoprecipitation. As previously reported, ZM treatment could reduce the level of phosphorylated form of hCAP-H in mitotic cells [25]. We found that hCAP-H co-immunoprecipitated with KIF4, either in the presence or absence of Aurora B activity (Fig 3D), suggesting that the interaction between KIF4 and condensin I is independent from phosphorylation by Aurora B.

**Fig 4:**
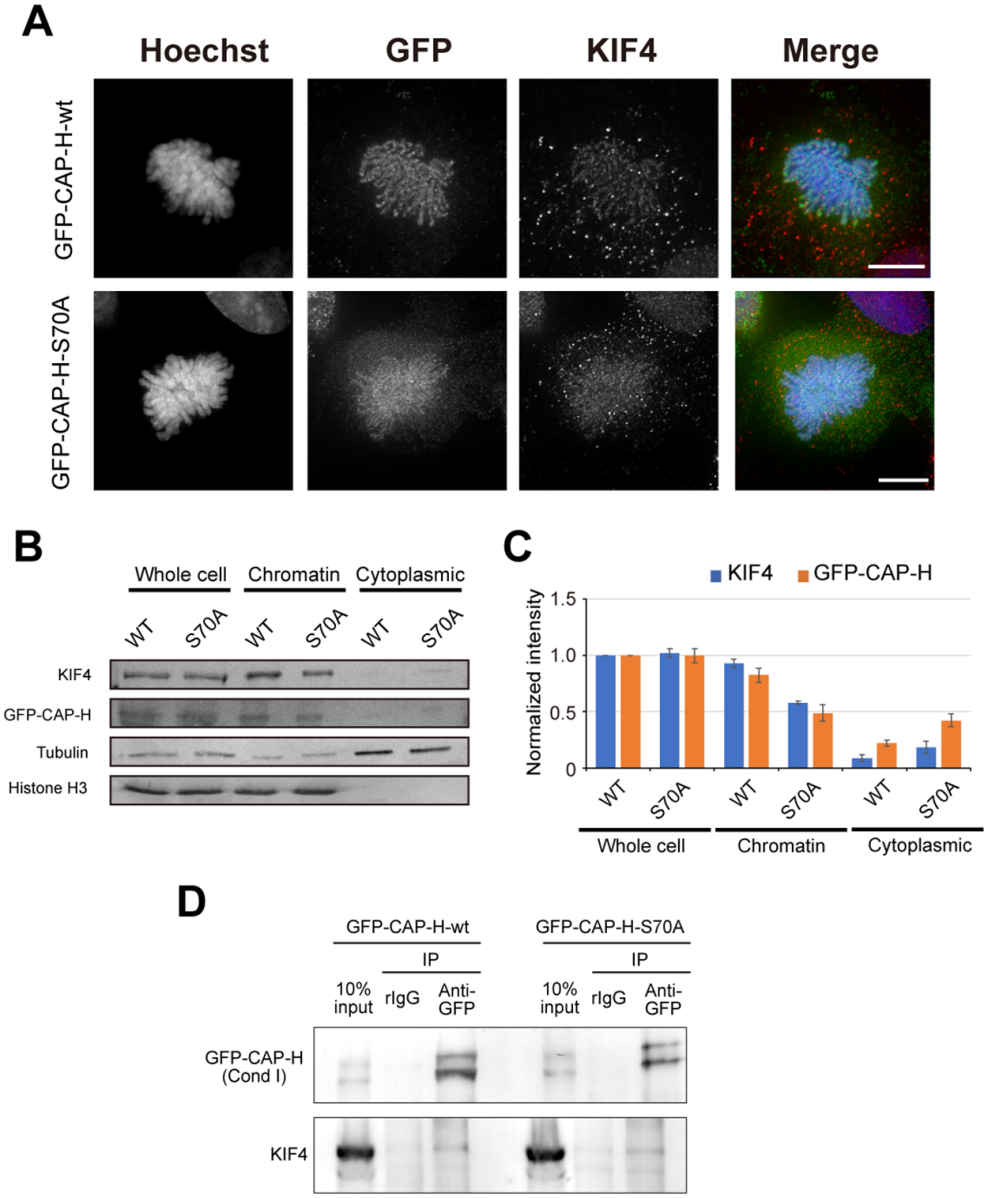
Interaction between KIF4 and condensin I is independent on Aurora B kinase activity. (A) HEK293T cells expressing GFP-hCAPH-wt or GFP-hCAPH-S70A were immunostained and z-stack images were obtained. After deconvolution process, projection images were prepared. Scale bar, 10 μm. (B) Western blot analysis of whole cell extract, chromatin fraction and cytoplasmic fraction prepared from mitotic HEK293T cells expressing GFP-hCAPH-wt or GFP-hCAPH-S70A. Tubulin and histone H3 were used as loading controls. (C) The band intensity of (B) was quantified using ImageJ software and normalized against protein amount in whole cell extract of GFP-hCAP-H-wt expressing cells. n = 3. (D) Physical interaction between GFP-hCAP-H-wt or GFP-hCAP-H-S70A and KIF4 in HEK293T cells. GFP-tagged hCAP-H were immunoprecipitated from chromatin-bound protein fractions of the cells as indicated. Co-immunoprecipitation of GFP-hCAP-H-wt or GFP-hCAP-H-S70A with KIF4 was analyzed by western blot. n = 3.

Tada *et al*. showed that S70 phosphorylation of hCAP-H by Aurora B largely facilitates loading of condensin I to chromosomes, however, a small amount of hCAP-H-S70A mutant were detected on the chromosomes [25]. To further confirm our previous observation that phosphorylation by Aurora B is not necessary for the interaction between KIF4 and condensin I, interactions of KIF4 with phosphorylated and unphosphorylated forms of hCAP-H were analyzed. First, we generated HEK293T cells expressing GFP-hCAP-H-wt or GFP-hCAP-H-S70A (Fig 4). In HEK293T expressing GFP-hCAP-H-wt, we found axial localization of GFP-hCAP-H-wt and KIF4 on chromosomes (Fig 4A). In HEK293T expressing GFP-hCAP-H-S70A, we detected GFP-hCAP-H-S70A as well as KIF4 on chromosomes. However, the localization of both GFP-hCAP-H-S70A as and KIF4 were diffused from chromosome to cytoplasm (Fig 4A–4C). This confirms defects in chromatin binding of hCAP-H-S70A mutant as previously reported [25]. This data also suggests that loss of hCAP-H on the chromosomes induces delocalization of KIF4, consistent with our previous results showing interdependency of condensin I and KIF4 on loading to chromosome scaffold (Fig 2).

Next, to check whether GFP-hCAP-H-S70A mutant interacts with KIF4, chromatin-bound protein fractions from HEK293T cells expressing GFP-hCAP-H-wt or GFP-hCAP-H-S70A were extracted and used for immunoprecipitation. The results showed that KIF4 co-immunoprecipitated with both GFP-hCAP-H-wt and GFP-hCAP-H-S70A (Fig 4D). This demonstrates that the interaction between KIF4 and condensin I on the chromosome is independent of hCAP-H phosphorylation at S70 by Aurora B. However, other phosphorylation sites may possibly be required for this interaction.

### Inhibition of Plk1 increases loading of KIF4 and condensin I to the chromosome

Plk1 is another kinase that plays important roles during cell division. Previous phosphoproteome analysis showed that KIF4 is a Plk1 substrate [29]. In addition, Plk1 has been proposed to function in opposition to Aurora B during the cell cycle [30]. This hypothesis suggests that Plk1 may regulate KIF4 and condensin I loading to chromosomes.

To address the function of Plk1-mediated phosphorylation, mitotic arrested HeLa cells were incubated in the absence or presence of Plk1 kinase inhibitor, BI2536 (BI) [31]. We found that accumulation of KIF4 and hCAP-H increased on chromosomes in the presence of BI when compared with that of the control (Fig 3B, 3C, 3E and 3H). These results suggest that Plk1 negatively regulates the loading of both KIF4 and condensin to the chromosome.

However, in KIF4 knockdown cells, BI treatment did not largely increase the amount of hCAP-H on the chromosome (Fig 3F and 3I), compared to mock treatment (Fig 3E and 3H). Reciprocally, in the absence of condensin I, BI treatment also did not largely increase the amount of KIF4 on the chromosome (Fig 3G and 3J, compared to mock treatment (Fig 3E and 3H). These results indicate an interdependency between KIF4 and condensin I is required for the accumulation of KIF4 and condensin I on chromosomes in the absence of Plk1 activity.

## Discussion

Our study demonstrated two important points for organization of the chromosome scaffold during mitosis. First, cooperation between scaffold proteins, such as the interdependency between KIF4 and condensin I is crucial for the formation of the proper chromosome scaffold structure. Second, regulation of scaffold protein loading to chromosomes by phosphorylation is critical for establishing the chromosome scaffold.

In this study, interdependency and physical interaction between KIF4 and condensin I for localization to the chromosome were revealed. These results suggest that the interaction between KIF4 and condensin I may assist both proteins to bind and organize stably to the chromosome scaffold. Fluorescence recovery after photobleaching (FRAP) experiments have shown that condensin I and KIF4 are dynamically exchanging between the chromosome and cytoplasm, and there are small immobilized fractions of these proteins on chromosomes [9, 24]. These previous observations imply that most of KIF4 and condensin I dynamically change their localization in the chromosome scaffold. Thus, we postulate that to ensure localization on the chromosome scaffold, KIF4 and condensin I would have to interact to form a stable complex. This stable complex is able to localize securely on the chromosome scaffold. Takahashi *et al*. suggested KIF4 and condensin I binding ratio is 2:1 [11]. However, our previous proteome analysis showed that the amounts of KIF4 and condensin I present on isolated chromosomes were practically comparable [32], suggesting that KIF4 and condensin I bind to each other at a 1:1 ratio on the chromosome. Because the binding ability of condensin I to KIF4 is different among condensin I subunits (hCAP-G, hCAP-D2 and hCAP-H) [11], the molecular ratio of stable functional KIF4/condensin I complex still remains to be answered.

KIF4 did not interact with condensin II in this study, consistent with the previous observation in *Xenopus* [15]. In this previous study, it was found that PP2A recruits both KIF4 and condensin II to chromosomes *via* a non-catalytic function. Based on the data, they suggested that KIF4 and condensin II are recruited independently to the chromosome via different PP2A subtypes. However, a recent report indicated that KIF4 interacts with PP2A-B560γ [33], similar to condensin II [15]. Thus, the mechanism of how KIF4 and condensin II are recruited to the chromosome by non-catalytic PP2A would require further investigation.

Aurora B and Plk1 kinase activities are required for regulation of chromosome attachment to spindle fibers and cytokinesis; although, these two kinases have antagonistic functions in some processes [30]. On the other hand, the functions of Aurora B and Plk1 in chromosome condensation remain unresolved. In this study, we demonstrated that inhibition of Aurora B and Plk1 resulted in decreases and increases of KIF4 and condensin I on chromosomes, respectively (Fig 3). These results would indicate that the antagonistic activities of Aurora B and Plk1 are also conserved in chromosome formation, and Aurora B and Plk1 function as positive and negative regulators, respectively, in the recruitment of KIF4 and condensin I to the chromosome.

Phosphorylation sites on KIF4 and condensin I that regulate their association or dissociation on the chromosome have not been determined in this study. However, previous phosphoproteome analysis has suggested possible phosphorylation sites on KIF4 and some subunits of condensin I by Aurora B and Plk1 [29]. S70 on hCAP-H of condensin I is an Aurora B-dependent phosphorylation site, which regulates condensin I localization to the chromosome [25]. Our results showed that the phosphorylation hCAP-H-S70 by Aurora B is not required for the interaction with KIF4; however, other phosphorylation sites should also be required for this interaction. KIF4 has a phosphorylation site by Aurora B at T799, which was shown to control KIF4 function during cytokinesis [34]; although, the study did not report the function of T799 phosphorylation for chromosome recruitment of KIF4. Interestingly, Takahashi *et al*. reported that the phosphorylation of KIF4 by Cdk1 and Aurora B is required for KIF4 interaction with condensin I [28]. This is inconsistent with our results showing phosphorylation by Aurora B is not important for the interaction between condensin I and KIF4. This difference might be caused by cell culture conditions and the antibody types used for IP. Additionally, in this previous study, although the interaction between KIF4 and condensin I was reduced, KIF4 still bound to condensin I. This suggests that Aurora B activity is not a main factor for the KIF4-condensin I interaction. Our results clearly showed that phosphorylation is a critical factor to control localization of KIF4 and condensin I to the chromosome, leading to proper chromosome scaffold construction, which subsequently promotes proper chromosome organization processes. However, the function of KIF4 phosphorylation by Aurora B for the interaction remains controversial.

Based on our present data, the dynamics of KIF4 and condensin I localization on the chromosome is proposed as follows. To recruit KIF4 and condensin I to the chromosome, Aurora B phosphorylates KIF4 and condensin I to stimulate their chromatin binding activity. Due to dynamic exchanges of KIF4 and condensin I between the chromosome and cytoplasm, KIF4 and condensin I then have to form a large stable complex to confine and secure their localization on the chromosome scaffold. On the other hand, chromatin binding of KIF4 and condensin I is also antagonistically regulated by Plk1, in which phosphorylation by Plk1 appears to block the association of KIF4 and condensin I to chromosomes or induces the dissociation of KIF4 and condensin I from the chromosome in the later stages of mitosis. In this study, although we reveal that interaction and phosphorylation of scaffold proteins such as condensin I and KIF4, could affect their localization on chromosome, how chromosome scaffold structure is changed by these regulations still remains to be resolved by high resolution imaging analysis/super-resolution microscopy.

## Supporting information

S1 Table List of antibody used in this study. The names of antigen, host, company name, catalog number and dillution rate of antibodies used in this study are indicated.
(DOCX)
