# Supplementary material for: Interdependency and phosphorylation of KIF4 and condensin I are essential for organization of chromosome scaffold
Source: PLoS One. 2017 Aug 17;12(8):e0183298. doi: 10.1371/journal.pone.0183298 (PMC5560531; doi:10.1371/journal.pone.0183298)
Supplement: S1 Table — The names of antigen, host, company name, catalog number and dillution rate of antibodies used in this study are indicated. (DOCX) [file pone.0183298.s001.docx]

| **Supplementary Table. List of antibody used in this study** | | | | |  |
| --- | --- | --- | --- | --- | --- |
|  |  |  |  |  |  |
| Name | Host | Company | Cat. No. | Targeting sequence | Dilution |
| SMC2 | Rabbit | Hokkaido System Science |  | KSKAKPPKGAHVEVC | 1:300 (IF), 1:1000 (WB) |
| hCAP-H | Rabbit | OPERON |  | GTEDLSDVLVRQGD | 1:100 (IF), 1:1000 (WB) |
| hCAP-D2 | Rabbit | OPERON |  | TTPILRASARRHRS | 1:1000 (WB) |
| hCAP-D3 | Rabbit | Sigma |  | SRRSLRKTPLKTA | 1:1000 (WB) |
| KIF4 | Rabbit | Thermo Fisher Scientific | PA5-30492 |  | 1:500 (IF), 1:1000 (WB) |
| GFP | Chicken | Abcam | ab13970 |  | 1:500 (IF) |
| CREST | Human | CORTEX | CS1058 |  | 1:500 (IF) |
| Histone H3 | Mouse | Upstate | 05-499 |  | 1:1000 (WB) |
| GFP | Rabbit | MBL | 598 |  | 1 μl/sample (IP) |
| Alpha-tubulin | Mouse | CALBIOCHEM | CP06 |  | 1:1000 (WB) |
| Anti-Human IgG TRITC conjugated | Goat | Sigma | T5903 |  | 1:1000 (IF) |
| Anti-Mouse IgG Alexa488 | Goat | Invitrogen | A11001 |  | 1:1000 (IF) |
| Anti-Rabbite IgG Alexa594 | Goat | Invitrogen | A11012 |  | 1:1000 (IF) |
| Anti-Mouse IgG Alexa594 | Goat | Invitrogen | A11005 |  | 1:1000 (IF) |
| Anti-chicken IgG Alexa488 | Goat | Invitrogen | A11039 |  | 1:1000 (IF) |
| Anti-Rabbite IgG Alexa488 | Goat | Invitrogen | A11008 |  | 1:1000 (IF) |
| Anti-rabbit IgG Alkaline Phosphatase | Goat | Vector Laboratories | AP-1000 |  | 1:3000 (WB) |
| Anti-mouse IgG Alkaline Phosphatase | Horse | Vector Laboratories | AP-2000 |  | 1:3000 (WB) |
